# Metabolic Constants and Plasticity of Cancer Cells in a Limiting Glucose and Glutamine Microenvironment—A Pyruvate Perspective

**DOI:** 10.3389/fonc.2020.596197

**Published:** 2020-12-08

**Authors:** Angela M. Otto

**Affiliations:** Munich School of BioEngineering, Technical University of Munich, Garching, Germany

**Keywords:** nutrient deprivation, ^13^C-glucose tracing, glycolysis, glutamine, TCA-cycle, anaplerosis, pyruvate replenishment, metabolic network

## Abstract

The metabolism of cancer cells is an issue of dealing with fluctuating and limiting levels of nutrients in a precarious microenvironment to ensure their vitality and propagation. Glucose and glutamine are central metabolites for catabolic and anabolic metabolism, which is in the limelight of numerous diagnostic methods and therapeutic targeting. Understanding tumor metabolism in conditions of nutrient depletion is important for such applications and for interpreting the readouts. To exemplify the metabolic network of tumor cells in a model system, the fate ^13^C_6_-glucose was tracked in a breast cancer cell line growing in variable low glucose/low glutamine conditions. ^13^C-glucose-derived metabolites allowed to deduce the engagement of metabolic pathways, namely glycolysis, the TCA-cycle including glutamine and pyruvate anaplerosis, amino acid synthesis (serine, glycine, aspartate, glutamate), gluconeogenesis, and pyruvate replenishment. While the metabolic program did not change, limiting glucose and glutamine supply reduced cellular metabolite levels and enhanced pyruvate recycling as well as pyruvate carboxylation for entry into the TCA-cycle. Otherwise, the same metabolic pathways, including gluconeogenesis, were similarly engaged with physiologically saturating as with limiting glucose and glutamine. Therefore, the metabolic plasticity in precarious nutritional microenvironment does not require metabolic reprogramming, but is based on dynamic changes in metabolite quantity, reaction rates, and directions of the existing metabolic network.

## Introduction—Cancer Cells in a Limiting Nutritional Microenvironment

**Figure 1:**
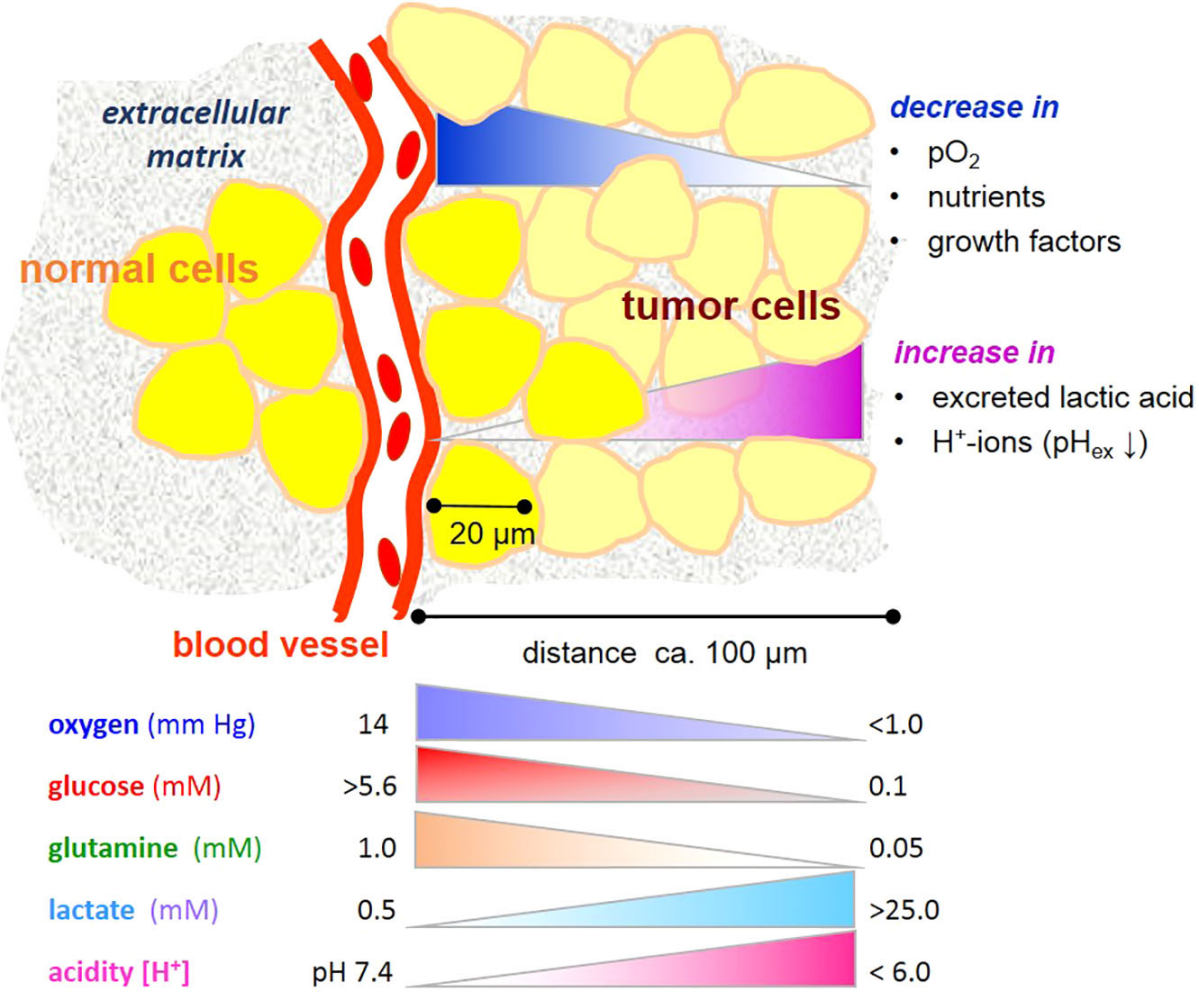
Gradients microenvironmental variables affecting metabolism of tumor cells. Note that the gradients are unlikely to be linear. Values are taken from references (1, 2).

Cancers consist of a plethora of cells with different metabolic phenotypes in a microenvironment of fluctuating nutrient availability, along with other changes in physico-chemical variables supporting or jeopardizing cell survival (**Figure 1**). This cellular heterogeneity poses a challenge for metabolic imaging, diagnosis and chemo-therapeutic targeting in the clinic. It is also a challenge for designing analytical conditions, namely, appropriate cell cultivation systems laid out for drug screening and preclinical testing. It is thus imperative from different perspectives to understand how the metabolism of cancer cells responds to changes in a precarious microenvironment (3).

Considering that numerous views and reviews have been written on cancer metabolism (3–6), each highlighting a different angle of the biochemistry and molecular biology of uncontrollable growth of cancer cells—which new perspective will be taken here? Our basic understanding of cancer metabolism has been shaped by studying cancer cells cultured mainly in *saturating* nutrient conditions, i.e. those resembling a well-fed, even diabetic physiology. However, metabolic plasticity of cancer cells is a feature for adapting to varying, often limiting levels of nutrients. How does a limiting supply of nutrients affect cancer metabolism? Is it a matter of metabolite quantity or of metabolic reprogramming?

In physiological conditions, blood contains about 0.6 mM glutamine and 5–11 mM glucose, depending on the nutritional state (7, 8). Within most cancers, and also in many normal tissues, these levels markedly decrease with increasing distance from a blood vessel and consumption by neighboring cells (**Figure 1**). On the other hand, cells may release metabolic products, e.g. amino acids and lactate, useful to neighboring cells. Even though the “Warburg effect”, i.e. aerobic glycolysis producing lactate, is considered to be one of the hallmarks of cancer (9), it is not unique to tumor cells, being also typical of several other cell types with high energy metabolism and/or proliferative propensity (10). Moreover, deviant glycolytic activity leading to excess lactate production is not the only inception for metabolic rearrangement in tumor cells. Metabolic plasticity is a survival trait for tumor cells in a microenvironment becoming increasingly depleted of nutrients. Few studies have addressed effects of glucose and glutamine deprivation in this context, and these indeed show that the limitation of *either* glucose or glutamine has profound effects on cancer cell growth (11), depending on the cell type and its oncogenic genotype, such as the expression of the tumor suppressor gene CC3/TIP30 (12), c-myc expression (13), and K-ras transfection (14). Also, a misbalance of glucose and glutamine levels adversely impacts glycolysis and can jeopardize cell viability (15), as will be further elucidated below.

The question here is thus: how does the metabolic phenotype change in tumor cells when *both* glucose and glutamine are limiting, as may occur in a precarious tumor microenvironment? In this review, I will track the fate of ^13^C_6_-labeled glucose and its derived metabolites in conditions of different levels of glutamine in MCF-7 cells, a low-malignant cell line expressing estrogen and progesterone receptors as well as the oncogenes p53 and c-myc (16, 17). Metabolite detection by gas chromatography with mass spectrometry (GC-MS) allows to identify and semi-quantify a number of metabolites with regards to 1) their cellular levels; 2) their ^13^C-enrichment, which is a product of the metabolite pool and metabolic flux; and 3) ^13^C-isotope profiles, which provide information on different metabolic roads taken by ^13^C-glucose-derived metabolites (18–21). These analyses attest pyruvate a key role in metabolic plasticity.

## The Glycolytic Road to the Pyruvate Junction

### Glucose and Glutamine—A Metabolic Affair

The degradation of glucose to pyruvate requires nine enzymatic steps. Firstly, however, glucose must be taken up by the cell. MCF-7 cells express the glucose transporters GLUT1 and GLUT3, whose levels are increased in hypoglycemic conditions and are rate limiting for glycolysis (22). GLUT1 is prominent in most cells, having a K_M_ in the range of 2–3 mM in MCF-7 cells (23). The high affinity of this transporter ensures that low glucose is taken up avidly. The first intracellular enzyme encountered is hexokinase (K_M_ = 0.1 mM), which rapidly phosphorylates glucose and is one of the flux-controlling enzymes of glycolysis (24). Maximal glycolytic activity is thus initiated with glucose well below normal physiological blood concentrations. The glycolytic flux is further regulated by the rate-limiting enzymes phosphofructokinase and, ultimately, monocarboxylate transporter 4 (MCT4) (24), responsible for expelling excess lactate out of the cell (25, 26). The observation that the expression of lactate dehydrogenase (LDH) did not show a flux-regulating effect is supported by data showing that its activities correlate poorly with other glycolytic parameters [see below (15, 27)].

Even though glutamine is not an essential amino acid for human cells, its extracellular level has a profound influence on glucose metabolism (28), and the *balance* of extracellular glucose to glutamine is a fundamental metabolic parameter (15). In cell cultures, the amount of glucose removed from the medium is a measure for cellular consumption, which depends on glucose availability as well as the cell’s metabolic demand; and this is co-dependent on the concentration of glutamine. Compared to other limiting conditions, MCF-7 cells were more viable with a balanced combination of low glutamine (0.1 mM) and 2.5 mM glucose than with the misbalanced combination of saturating glutamine (1 mM) and 1 mM glucose; the latter attenuated glycolytic activities, reduced NAD+/NADH ratio, and resulted in cell death (15). Therefore, coherent with a similar observation with MDA-MB231 cells (14), glutamine could not rescue these cells from glucose deprivation. Thus, an imbalance of glycolysis and glutaminolysis invokes metabolic distress.

### Turnoff to Serine and Glycine Synthesis

Along the glycolytic path, 3-phosphoglycerate is at the turn off to serine and glycine synthesis, which is intimately related to glycolytic activity (29). The extracellular level of serine (in cell culture medium) is usually 400 µM, and thus saturating for its transporter ASCT2, which has a K_M_ of approximately 20 µM (30); intracellular and extracellular concentrations are reported to be in equilibrium (31). Nevertheless, the *intracellular* levels of serine and glycine in MCF-7 cells varied depending also on the combinations of low glucose/glutamine. Notably, the serine pool was not any higher with 25 mM than with 2.5 mM glucose. However, the ^13^C-labeling of serine and glycine from ^13^C_6_-glucose indicates that they are also synthesized *de novo*, albeit at a very low level of 1%–3% (27), which may be essential for one-carbon metabolism (29). Moreover, the enrichment of ^13^C-glycine stands out in that it is 3–5-fold higher in the saturating than in low glucose conditions, which may reflect the enhanced requirements for one-carbon metabolism of proliferative cells in high glucose/glutamine conditions. Altogether, these results illustrate how the availability of glucose and glutamine affect serine synthesis (32).

### The Pyruvate Junction

**Figure 2:**
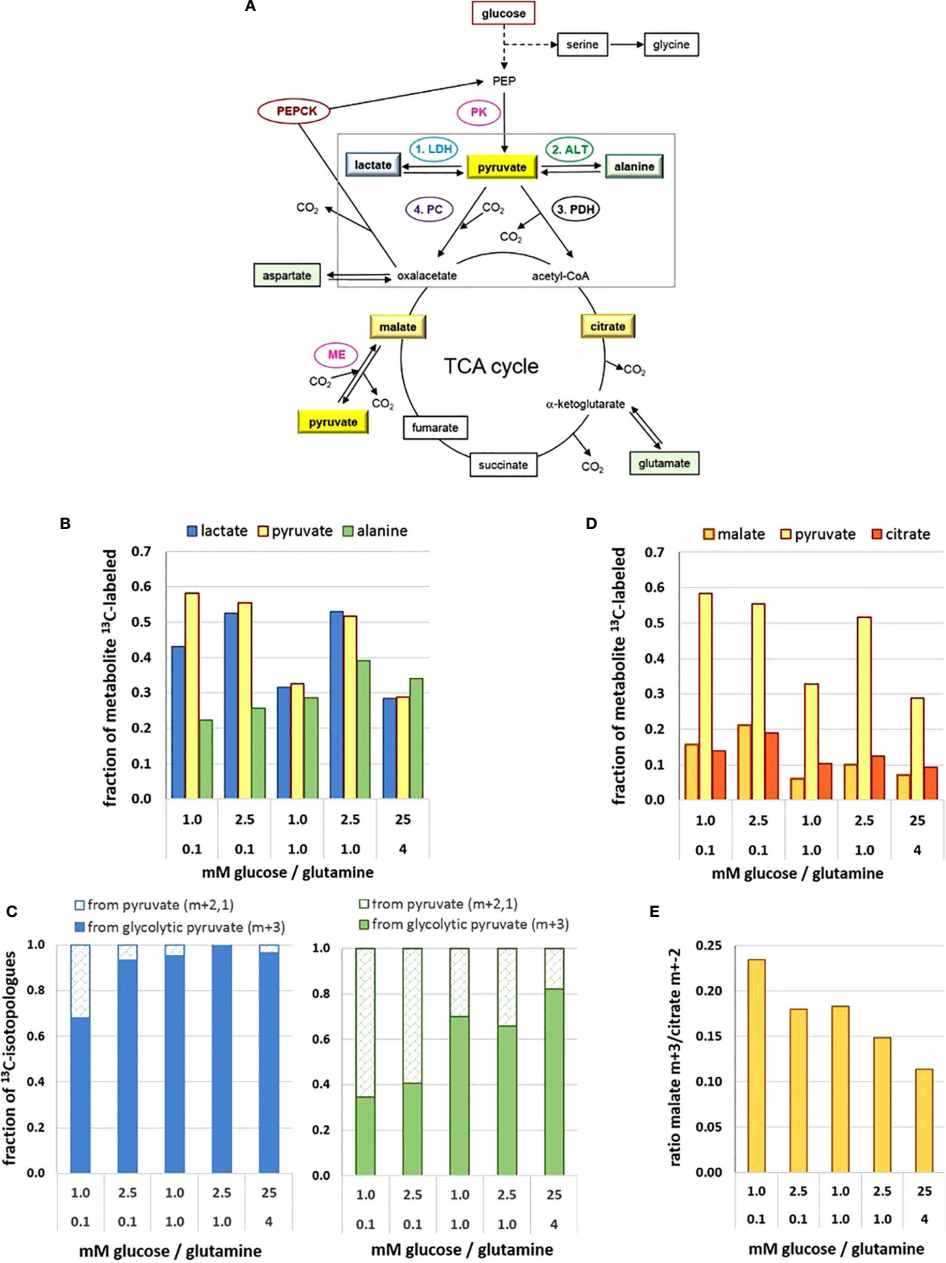
Metabolic flux and pathways to and from the pyruvate junction depend on glucose/glutamine concentrations. **(A)** Pathways of ^13^C-glucose-derived metabolites and the pyruvate junction. **(B)** ^13^C-enrichment of pyruvate, lactate, and alanine pools; **(C)** changes in the fraction of ^13^C-isotopologue profiles of lactate (left) and alanine (right) derived from either glycolytic (m+3) or replenished (m+2,1) ^13^C-pyruvate; **(D)** ^13^C-enrichment of pyruvate, malate and citrate pools; **(E)** changes in the ratio of ^13^C_3_-pyruvate carboxylation versus decarboxylation as indicated by the ^13^C-malate m+3/^13^C-citrate m+2 ratio. The figure is based on data obtained following a 2h ^13^C_6_-glucose incubation, retrieved and calculated from (27). Detected ^13^C-labeled metabolites are framed. ALT, alanine aminotransferase; LDH, lactate dehydrogenase; ME, malic enzyme; PDH, pyruvate dehydrogenase complex; PC, pyruvate carboxylase; PK, pyruvate kinase.

The end product of glycolysis is pyruvate (**Figure 2A**). The final enzymatic step converting phosphoenol pyruvate (PEP) to pyruvate is controlled by pyruvate kinase (PK), the reaction being virtually irreversible due its ATP production. Increased expression of the PKM2 isoform is found in many cancer cells, albeit often in its dimeric less active state (33); and being an allosteric enzyme, several metabolites regulate its activity, including alanine and serine (29, 34). In extracts of MCF-7 cells, PK activity was about 2-3 fold lower in limiting than in saturating glucose/glutamine conditions (27). However, cells in low (2.5. mM) glucose had a higher PK activity with 0.1 mM than with 1 mM glutamine. This is again evidence for glutamine in misbalance with glucose suppressing glycolysis (15).

In comparison to a range of other glucose-derived metabolites in MCF-7 cells, the level of pyruvate in cell extracts was remarkably low, estimated at 10–100 µM (35), with little variation in the different *limiting* conditions, but being about 3-fold higher in the 25 mM glucose conditions (27). However, upon incubation with ^13^C_6_-glucose, ^13^C-enrichment of pyruvate amounted up to about 60% in the different low glucose/glutamine conditions (**Figure 2B**), with the exception of the misbalanced 1 mM glucose/1 mM glutamine combination, where ^13^C-enrichment was only about 30%. The unexpectedly low ^13^C-enrichment with 25 mM ^13^C_6_-glucose is explained by a high intracellular metabolite level and thus limited glucose uptake. The differences in ^13^C-enrichment values corresponded well with the respective PK activities (27). Thus at the terminus of glycolysis, enrichment of ^13^C_3_-pyruvate reflects differences in glycolytic flux resulting from differences in low glucose and glutamine concentrations.

## Metabolic Decisions at the Pyruvate Junction

Pyruvate constitutes a metabolic junction with different options: 1) NADH-mediated reduction to lactate, 2) glutamine-mediated amination to alanine, 3) carboxylation to oxaloacetate, and 4) decarboxylation to acetyl-CoA (**Figure 2A**). The road taken will depend on the metabolic needs of the cell, thereby endowing the cells with high metabolic plasticity.

### Conversion to Lactate

In cancer cells, most of the glycolytic pyruvate is rapidly converted to lactate in the cytosol, catalyzed by lactate dehydrogenase of type A (LDH-A). However, the metabolic differences of MCF-7 cells in the different glucose/glutamine conditions were not mirrored in their LDH-A activity. Moreover, in MCF-7 cells, activity of LDH-A was lower than that of pyruvate kinase—an observation which cannot be generalized for other cell types. Indeed, LDH-A is not considered to be a flux controlling enzyme in glycolysis and for lactate export (24). Instead, MCT4 appears to be the controlling element for lactate release (26) and responsible for maintaining a constant intracellular lactate level. The amounts of lactate released in the different conditions (within 72 h) showed only small variations, ranging from about 0.5 to 0.8 µmol/10e5 cells for media initially containing 1 to 5.6 mM glucose, respectively (27); this amounted to an accumulation of lactate between 1 and 5 mM, increasing with the cell number. Thus, lactate release is not affected by its extracellular level, but rather managed by the efflux activity of MCTs (26).

In the *intracellular* compartment, the level of lactate was about 45- to 70-fold higher than that of pyruvate, depending on the glucose/glutamine combination. Lactate maintained a basal level of about 2–4 mM, showing little variation with respect to different limiting glucose/glutamine conditions, with the exception of temporal increases up to 20 mM with the balanced combination of 2.5 mM glucose/0.1 mM glutamine. Also, in saturating conditions (25 mM glucose) the basal lactate level of MCF-7 cells was enhanced up to about 40 mM (27), which might be explained by the saturation of export capacity for lactate in relation to its production rate. Notably, there appears to be no direct quantitative relationship between available glucose and the corresponding *intracellular* lactate levels.

The ^13^C-enrichment of ^13^C-lactate was very similar to that of ^13^C-pyruvate, as to be expected for its rapid turnover by LDH-A (**Figure 2B**). This suggests that pyruvate is a metabolite which is not stored by the cell and fosters the idea that lactate serves as a reservoir for rapidly providing pyruvate when needed (27). The ^13^C-tracer experiments indicate that the main flux of ^13^C-pyruvate was to ^13^C- lactate, but this depended on the levels of glucose and glutamine. Moreover, analyzing the ^13^C- isotopologues m+3,2,1 (m = number of ^13^C per molecule) showed that in standard high glucose conditions, 99% of ^13^C-lactate was m+3, that is, derived from ^13^C_3_ –pyruvate directly from glycolysis (denoted here as “glycolytic pyruvate”). In contrast, in the most precarious conditions (1 mM glucose/0.1 mM glutamine) only 75% of ^13^C_3_- lactate came from glycolytic pyruvate (**Figure 2C**). The increased fraction of the pyruvate and lactate isotopologues with m+2,1, however, indicates that these molecules were no longer a direct product of glycolysis. Instead, ^13^C_2_-pyruvate can result from a 1. round of the TCA-cycle providing ^13^C_2_-malate, which is decarboxylated to ^13^C_2_-pyruvate *via* the mitochondrial malic enzyme (36). Isotopologues of m+1 can also evolve depending on the availability of ^13^C-pyruvate or ^13^C-oxaloacetate in further TCA-cycles. Either way, glycolytic pyruvate can be replenished *via* the TCA-cycle.

### Conversion to Alanine

The direct amination of pyruvate *via* the (glutamine-dependent) alanine aminotransferase (ALT) leads to alanine. Its cellular levels were generally much lower than those of lactate, but very sensitive to different low glucose/glutamine concentrations; also, alanine was 10-fold higher in the standard than the most limiting glucose conditions (27). Remarkably, ^13^C- enrichment of alanine derived from ^13^C_6_-glucose did not reflect that of ^13^C-pyruvate, as might be expected from a direct amination reaction (**Figure 2B**). With increasing nutrient availability ^13^C-alanine enrichment showed only moderate increases, implying that sufficient alanine levels could be achieved with *low* glucose and glutamine. Interestingly, in conditions with 0.1 mM glutamine, the ^13^C-enrichment kinetics of alanine (up to 20 h) resembled those of ^13^C-labeled TCA-metabolites (27), suggesting its conversion *via* the mitochondrial ALT2, which has high pyruvate affinity (37). At higher glutamine concentrations, however, ^13^C-alanine flux resembled that of pyruvate and lactate, suggesting a cytosolic conversion by ALT1. The isotopologues profiles of alanine with a low fraction of m+3, particularly in limiting glutamine conditions (**Figure 2C**), suggests that glycolytic pyruvate may *not* be the major precursor for alanine (**Figure 2C**), as will be discussed further below.

### Decarboxylation to Acetyl-CoA

The most efficient way for pyruvate serving the cells’ energy and anabolic needs is its entry into the TCA cycle (**Figure 2A**). The mitochondrial import carrier (MPC) has a K_M_ for pyruvate of about 150 µM (38), and for its decarboxylation the allosteric enzyme complex pyruvate dehydrogenase (PDH) has a K_M_ of 17–36 µM (39), suggesting that pyruvate uptake is not rate-limiting for PDH activity. The level of citrate was found to be similarly low as that of pyruvate, being almost invariable in the different conditions (27). In contrast, the flux of ^13^C- metabolites in the TCA cycle was both glucose and glutamine dependent (**Figure 2D**), ^13^C-enrichment being up to 20% with low glucose/glutamine conditions, but < 10% with saturating glutamine conditions. Such reduced ^13^C-levels were most evident for ^13^C-glutamate, ^13^C-succinate, ^13^C-fumarate, ^13^C-malate and ^13^C-aspartate, indicative of glutamine anaplerosis *via* alpha-ketoglutarate. Moreover, similarities in glucose/glutamine dependent ^13^C-enrichment patterns for these metabolites suggest that there is no reprogramming of the canonical progression through the TCA-cycle, but rather a change in the flux rate depending on metabolite levels. Analyzing the ^13^C- isotopologues from the 1. round TCA-cycle revealed that in the various limiting conditions about 35%–48% of citrate (m+2) came from glycolytic pyruvate (m+3), while in saturating conditions the utilization of the glycolytic pyruvate for citrate exceeded 60% (27). This increased demand for glycolytic pyruvate could be explained by a higher metabolic flux for oxidative and anabolic metabolism to support enhanced cell proliferation.

### Carboxylation to Oxaloacetate

Another function of pyruvate is to feed the TCA-cycle by being carboxylated to oxaloacetate *via* the mitochondrial pyruvate carboxylase (PC), thereby also contributing the precursor for its acetylation to citrate as well as for other metabolic pathways (40). Since oxaloacetate was not detectable in the experimental setup, ^13^C- glucose-derived oxalate was estimated indirectly *via* ^13^C-malate, (**Figure 2D**) (27). ^13^C-enrichment of malate was almost 20% in conditions with 0.1 mM glutamine, in contrast to ≤ 10% for cells with higher glutamine levels, reflecting the influence of glutamine anaplerosis. However, isotopologue profiling, showed that ^13^C-malate had not only the expected m+2 signature, but also a higher fraction of m+3 isotopologues than ^13^C-succinate. This indicates carboxylation of glycolytic ^13^C_3_-pyruvate (**Figure 2A**).

Both pyruvate carboxylation and decarboxylation taking place in the mitochondria calls for a metabolic decision. In this cellular model, the ^13^C-isotopologue profiles of citrate and malate revealed that pyruvate decarboxylation to acetyl-CoA was preferred over pyruvate carboxylation to oxaloacetate by a factor of about 4–10, whereby cells in limiting glucose/glutamine had a 2-fold higher preference for channeling glycolytic pyruvate to oxaloacetate than the satiated cells (**Figure 2E**). This illustrates how nutritional availability can change the port of entry for pyruvate into the TCA-cycle.

## Roundabouts for Glucose-Derived Metabolites

### Pyruvate Replenishment

Not only glycolytic ^13^C_3_-pyruvate but also ^13^C-pyruvate replenished from the TCA-cycle can serve as a substrate for ^13^C-alanine and ^13^C-lactate (27). The isotopologue fractions m+2,1 of ^13^C-lactate and ^13^C-alanine varied between 5% and 45%, depending on the glucose/glutamine conditions (**Figures 2C, D**). While over 95% of ^13^C-lactate came directly from glycolytic ^13^C-pyruvate in saturating conditions, this applied to only 70% in the most limiting glucose/glutamine conditions. Even more pronounced, in limiting glucose/glutamine conditions over 60% of ^13^C- alanine originated from replenished pyruvate (**Figure 2C**), while in saturating conditions, it was less than 20%. There are various reports suggesting a metabolic compartmentation of pyruvate (41, 42); the differential conversion of pyruvate supports such a concept. Moreover, the isotopologue profiles suggest that pyruvate comes from different metabolic backgrounds. Thus pyruvate metabolism is at the hub of metabolic plasticity.

### Gluconeogenesis

Recent work has established gluconeogenesis as a fundamental element of glucose metabolism in cancer cells, with the activity of phosphoenolpyruvate kinase (PEPCK) converting oxaloacetate to phosphoenolpyruvate (PEP), especially with glucose deprivation (43–46) (**Figure 2A**). Evidence for gluconeogenesis being involved in different nutrient conditions is provided by the isotopologue distribution of ^13^C-serine which, along with the expected glycolytic m+3, has an approximately 50% fraction of m+2,1 (27). This corroborates reports on gluconeogenesis contributing to serine synthesis (46, 47). The highest value of the combined m+2,1 fractions of over 70% was actually found with high glucose conditions, suggesting that the reversal of the glycolytic pathway is not reserved for precarious nutrient conditions.

## Concluding Thoughts

This narrative of tracking the fate of ^13^C-glucose illustrates how tumor cells in a microenvironment of fluctuating glucose and glutamine levels can employ a remarkably conservative metabolic program, but use it with high flexibility when rapidly adapting to limiting nutrient conditions. This adaptation employs dynamic modes in tuning metabolite levels, enzyme activities, flux rates, and bi-directionality of metabolic pathways. Here, pyruvate is a central metabolite for different metabolic paths, with pyruvate anaplerosis and replenishment having key roles by sustaining and regulating processes for a coordinated glucose and glutamine metabolism. Such metabolic plasticity constitutes a challenge for developing targeted and reliable diagnostic tools and therapeutic strategies in cancer.

## Author Contributions

The author confirms being the sole contributor of this work and has approved it for publication.

## Funding

The author’s work was funded by the German Research Council (2012.094.1) (DFG OT 92/3-2) and the Wilhelm Sander-Stiftung (2012.094.1), Munich, Germany.

## Conflict of Interest

The author declares that the research was conducted in the absence of any commercial or financial relationships that could be construed as a potential conflict of interest.

## References

[1] Kallinowski F Runkel S Fortmeyer HP Forster H Vaupel P L-glutamine: a major substrate for tumor cells in vivo? J Cancer Res Clin Oncol (1987) 113(3):209–15. 10.1007/BF00396375 PMC12248313 3584211

[2] Schaefer C Mayer WK Kruger W Vaupel P Microregional distributions of glucose, lactate, ATP and tissue pH in experimental tumours upon local hyperthermia and/or hyperglycaemia. J Cancer Res Clin Oncol (1993) 119(10):599–608. 10.1007/BF01372723 8335679 PMC12200585

[3] Vander Heiden MG DeBerardinis RJ Understanding the Intersections between Metabolism and Cancer Biology. Cell (2017) 168(4):657–69. 10.1016/j.cell.2016.12.039 PMC5329766 28187287

[4] Icard P Lincet H A global view of the biochemical pathways involved in the regulation of the metabolism of cancer cells. Biochim Biophys Acta - Rev Cancer (2012) 1826(2):423–33. 10.1016/j.bbcan.2012.07.001 22841746

[5] Alfarouk KO Ibrahim ME Gatenby RA Brown JS Riparian ecosystems in human cancers. Evol Appl (2013) 6(1):46–53. 10.1111/eva.12015 23396634 PMC3567470

[6] Reshkin SJ Cardone RA Harguindey S Na+-H+ Exchanger, pH Regulation and Cancer. Rec Pat Anti Canc Drug Dis (2013) 8(1):85–99. 10.2174/1574892811308010085 22738122

[7] Gullino PM Clark SH Grantham FH The interstitial fluid of solid tumors. Cancer Res (1964) 24:780–94. 14190544

[8] Felig P Wahren J Raf L Evidence of inter-organ amino-acid transport by blood cells in humans. Proc Natl Acad Sci USA (1973) 70(6):1775–9. 10.1073/pnas.70.6.1775 PMC433594 4515937

[9] Hanahan D Weinberg RA Hallmarks of cancer: the next generation. Cell (2011) 144:646–74. 10.1016/j.cell.2011.02.013 21376230

[10] Stark H Fichtner M Konig R Lorkowski S Schuster S Causes of upregulation of glycolysis in lymphocytes upon stimulation. A comparison with other cell types. Biochimie (2015) 118:185–94. 10.1016/j.biochi.2015.09.017 26382968

[11] Dang CV Glutaminolysis Supplying carbon or nitrogen or both for cancer cells? Cell Cycle (2010) 9(19):3884–6. 10.4161/cc.9.19.13302 20948290

[12] Chen V Shtivelman E CC3/TIP30 regulates metabolic adaptation of tumor cells to glucose limitation. Cell Cycle (2010) 9(24):4941–53. 10.4161/cc.9.24.14230 PMC3047816 21150275

[13] Yuneva M Zamboni N Oefner P Sachidanandam R Lazebnik Y Deficiency in glutamine but not glucose induces MYC-dependent apoptosis in human cells. J Cell Biol (2007) 178(1):93–105. 10.1083/jcb.200703099 17606868 PMC2064426

[14] Gaglio D Metallo CM Gameiro PA Hiller K Danna LS Balestrieri C Oncogenic K-Ras decouples glucose and glutamine metabolism to support cancer cell growth. Mol Syst Biol (2011) 7:523. 10.1038/msb.2011.56 21847114 PMC3202795

[15] Otto AM Hintermair J Janzon C NADH-Linked Metabolic Plasticity of MCF-7 Breast Cancer Cells Surviving in a Nutrient-Deprived Microenvironment. J Cell Biochem (2015) 116:822–35. 10.1002/jcb.25038 25530451

[16] Takahashi K Sumimoto H Suzuki K Ono T Protein-synthesis dependent cytoplasmic translocation of p53 protein after serum stimulation of growth-arrested MCF-7 cells. Mol Carcinogenesis (1993) 8(1):58–66. 10.1002/mc.2940080112 8352892

[17] Dubik D Dembinski TC Shiu RP Stimulation of c-myc oncogene expression associated with estrogen-induced proliferation of human breast cancer cells. Cancer Res (1987) 47(24 Pt 1):6517–21. 3677090

[18] Yang C Richardson AD Osterman A Smith JW Profiling of central metabolism in human cancer cells by two-dimensional NMR, GC-MS analysis, and isotopomer modeling. Metabolomics (2008) 4(1):13–29. 10.1007/s11306-007-0094-y

[19] Le A Lane AN Hamaker M Bose S Gouw A Barbi J Glucose-Independent Glutamine Metabolism via TCA Cycling for Proliferation and Survival in B Cells. Cell Metab (2012) 15(1):110–21. 10.1016/j.cmet.2011.12.009 PMC3345194 22225880

[20] Buescher JM Antoniewicz MR Boros LG Burgess SC Brunengraber H Clish CB A roadmap for interpreting 13C metabolite labeling patterns from cells. Curr Opin Biotechnol (2015) 34:189–201. 10.1016/j.copbio.2015.02.003 25731751 PMC4552607

[21] Dong W Keibler MA Stephanopoulos G Review of metabolic pathways activated in cancer cells as determined through isotopic labeling and network analysis. Metab Eng (2017) 43(Pt B):113–24. 10.1016/j.ymben.2017.02.002 PMC5552450 28192215

[22] Marín-Hernández A López-Ramírez SY Del Mazo-Monsalvo I Gallardo-Pérez JC Rodríguez-Enríquez S Moreno-Sánchez R Modeling cancer glycolysis under hypoglycemia, and the role played by the differential expression of glycolytic isoforms. FEBS J (2014) 281(15):3325–45. 10.1111/febs.12864 24912776

[23] Zamora-Leon SP Golde DW Concha II Rivas CI DelgadoLopez F Baselga J Expression of the fructose transporter GLUT5 in human breast cancer. Proc Natl Acad Sci U States America (1996) 93(5):1847–52. 10.1073/pnas.93.5.1847 PMC39870 8700847

[24] Tanner LB Goglia AG Wei MH Sehgal T Parsons LR Park JO Four Key Steps Control Glycolytic Flux in Mammalian Cells. Cell Syst (2018) 7(1):49–62.e8. 10.1016/j.cels.2018.06.003 29960885 PMC6062487

[25] Ganapathy V Thangaraju M Gopal E Martin PM Itagaki S Miyauchi S Sodium-coupled monocarboxylate transporters in normal tissues and in cancer. AAPS J (2008) 10(1):193–9. 10.1208/s12248-008-9022-y PMC2751467 18446519

[26] Contreras-Baeza Y Sandoval P Alarcón R Galaz A Cortés-Molina F Alegría K Monocarboxylate transporter 4 (MCT4) is a high affinity transporter capable of exporting lactate in high-lactate microenvironments. J Biol Chem (2019) 294:20135–47. 10.1074/jbc.RA119.009093 PMC6937558 31719150

[27] Gkiouli M Biechl P Eisenreich W Otto AM Diverse Roads Taken by 13C-Glucose-Derived Metabolites in Breast Cancer Cells Exposed to Limiting Glucose and Glutamine Conditions. Cells (2019) 8(10):1113. 10.3390/cells8101113 PMC6829299 31547005

[28] Dang CV Rethinking the Warburg Effect with Myc Micromanaging Glutamine Metabolism. Cancer Res (2010) 70(3):859–62. 10.1158/0008-5472.CAN-09-3556 PMC2818441 20086171

[29] Chaneton B Hillmann P Zheng L Martin ACL Maddocks ODK Chokkathukalam A Serine is a natural ligand and allosteric activator of pyruvate kinase M2. Nature (2012) 491(7424):458–62. 10.1038/nature11540 PMC3894725 23064226

[30] UtsunomiyaTate N Endou H Kanai Y Cloning and functional characterization of a system ASC-like Na+-dependent neutral amino acid transporter. J Biol Chem (1996) 271(25):14883–90. 10.1074/jbc.271.25.14883 8662767

[31] Possemato R Marks KM Shaul YD Pacold ME Kim D Birsoy K Functional genomics reveal that the serine synthesis pathway is essential in breast cancer. Nature (2011) 476:346. 10.1038/nature10350 21760589 PMC3353325

[32] Newman AC Maddocks ODK Serine and Functional Metabolites in Cancer. Trends Cell Biol (2017) 27(9):645–57. 10.1016/j.tcb.2017.05.001 28601431

[33] Mazurek S Grimm H Boschek CB Vaupel P Eigenbrodt E Pyruvate kinase type M2: a crossroad in the tumor metabolome. Br J Nutr (2002) 87:S23–S9. 10.1079/BJN2001454 11895152

[34] Jimenez de Asua L Rozengurt E Devalle JJ Carminatti H Some kinetic differences between the M isoenzymes of pyruvate kinase from liver and muscle. Biochim Biophys Acta (1971) 235(2):326–34. 10.1016/0005-2744(71)90211-7 5317638

[35] Zhang MM Pan YD Tang DH Dorfman RG Xu L Zhou Q Low levels of pyruvate induced by a positive feedback loop protects cholangiocarcinoma cells from apoptosis. Cell Communication Signaling (2019) 17:23. 10.1186/s12964-019-0332-8 30866966 PMC6417221

[36] Vacanti Nathaniel M Divakaruni Ajit S Green Courtney R Parker Seth J Henry Robert R Ciaraldi Theodore P Regulation of Substrate Utilization by the Mitochondrial Pyruvate Carrier. Mol Cell (2014) 56(3):425–35. 10.1016/j.molcel.2014.09.024 PMC4267523 25458843

[37] Glinghammar B Rafter I Lindstrom AK Hedberg JJ Andersson HB Lindblom P Detection of the mitochondrial and catalytically active alanine aminotransferase in human tissues and plasma. Intl J Mol Med (2009) 23(5):621–31. 10.3892/ijmm_00000173 19360321

[38] Halestrap AP The mitochondrial pyruvate carrier. Kinetics and specificity for substrates and inhibitors. Biochem J (1975) 148(1):85–96. 10.1042/bj1480085 1156402 PMC1165509

[39] Lazo PA Sols A Pyruvate dehydrogenase complex of ascites tumour. Activation by AMP and other properties of potential significance in metabolic regulation. Biochem J (1980) 190(3):705–10. 10.1042/bj1900705 PMC1162150 7193456

[40] Phannasil P Ansari I-u El Azzouny M Longacre MJ Rattanapornsompong K Burant CF Mass spectrometry analysis shows the biosynthetic pathways supported by pyruvate carboxylase in highly invasive breast cancer cells. Biochim Biophys Acta (BBA) - Mol Basis Dis (2017) 1863(2):537–51. 10.1016/j.bbadis.2016.11.021 PMC5243144 27890529

[41] Groen AK Sips HJ Vervoorn RC Tager JM Intracellular Compartmentation and Control of Alanine Metabolism in Rat Liver Parenchymal Cells. Eur J Biochem (1982) 122(1):87–93. 10.1111/j.1432-1033.1982.tb05851.x 7060572

[42] Zwingmann C Richter-Landsberg C Leibfritz D 13C isotopomer analysis of glucose and alanine metabolism reveals cytosolic pyruvate compartmentation as part of energy metabolism in astrocytes. Glia (2001) 34(3):200–12. 10.1002/glia.1054 11329182

[43] Leithner K Hrzenjak A Trotzmuller M Moustafa T Kofeler HC Wohlkoenig C PCK2 activation mediates an adaptive response to glucose depletion in lung cancer. ONCOGENE (2015) 34(8):1050–44. 10.1038/onc.2014.47 24632615

[44] Méndez-Lucas A Hyroššová P Novellasdemunt L Viñals F Perales JC Mitochondrial Phosphoenolpyruvate Carboxykinase (PEPCK-M) Is a Pro-survival, Endoplasmic Reticulum (ER) Stress Response Gene Involved in Tumor Cell Adaptation to Nutrient Availability. J Biol Chem (2014) 289(32):22090–102. 10.1074/jbc.M114.566927 PMC4139223 24973213

[45] Montal Emily D Dewi R Bhalla K Ou L Hwang Bor J Ropell Ashley E PEPCK Coordinates the Regulation of Central Carbon Metabolism to Promote Cancer Cell Growth. Mol Cell (2015) 60:571–83. 10.1016/j.molcel.2015.09.025 PMC4656111 26481663

[46] Vincent EE Sergushichev A Griss T Gingras MC Samborska B Ntimbane T Mitochondrial Phosphoenolpyruvate Carboxykinase Regulates Metabolic Adaptation and Enables Glucose-Independent Tumor Growth. Mol Cell (2015) 60(2):195–207. 10.1016/j.molcel.2015.08.013 26474064

[47] Yang M Vousden KH Serine and one-carbon metabolism in cancer. Nat Rev Cancer (2016) 16:650. 10.1038/nrc.2016.81 27634448

